# Prediction of tissue-specific effects of gene knockout on apoptosis in different anatomical structures of human brain

**DOI:** 10.1186/1471-2164-16-S13-S3

**Published:** 2015-12-16

**Authors:** Evgeny D Petrovskiy, Olga V Saik, Evgeny S Tiys, Inna N Lavrik, Nikolay A Kolchanov, Vladimir A Ivanisenko

**Affiliations:** 1 The Federal Research Center Institute of Cytology and Genetics, The Siberian Branch of the Russian Academy of Sciences, Prospekt Lavrentyeva 10, Novosibirsk, 630090, Russia; 2 International Tomography Center, The Siberian Branch of the Russian Academy of Sciences, Institutskaya 3A, Novosibirsk, 630090, Russia; 3 Otto von Guericke University Magdeburg, Medical Faculty, Department Translational Inflammation Research, Institute of Experimental Internal Medicine, Pfälzer Platz, Building 28, Magdeburg, 39106, Germany

**Keywords:** Gene expression, Machine learning, Regression models, Knockout effect prediction, Human brain, Drug target prediction, Neurodegenerative diseases

## Abstract

**Background:**

An important issue in the target identification for the drug design is the tissue-specific effect of inhibition of target genes. The task of assessing the tissue-specific effect in suppressing gene activity is especially relevant in the studies of the brain, because a significant variability in gene expression levels among different areas of the brain was well documented.

**Results:**

A method is proposed for constructing statistical models to predict the potential effect of the knockout of target genes on the expression of genes involved in the regulation of apoptosis in various brain regions. The model connects the expression of the objective group of genes with expression of the target gene by means of machine learning models trained on available expression data. Information about the interactions between target and objective genes is determined by reconstruction of target-centric gene network. STRING and ANDSystem databases are used for the reconstruction of gene networks. The developed models have been used to analyse gene knockout effects of more than 7,500 target genes on the expression of 1,900 objective genes associated with the Gene Ontology category "apoptotic process". The tissue-specific effect was calculated for 12 main anatomical structures of the human brain. Initial values of gene expression in these anatomical structures were taken from the Allen Brain Atlas database. The results of the predictions of the effect of suppressing the activity of target genes on apoptosis, calculated on average for all brain structures, were in good agreement with experimental data on siRNA-inhibition.

**Conclusions:**

This theoretical paper presents an approach that can be used to assess tissue-specific gene knockout effect on gene expression of the studied biological process in various structures of the brain. Genes that, according to the predictions of the model, have the highest values of tissue-specific effects on the apoptosis network can be considered as potential pharmacological targets for the development of drugs that would potentially have strong effect on the specific area of the brain and a much weaker effect on other brain structures. Further experiments should be provided in order to confirm the potential findings of the method.

## Background

At present time of intensive investigations of the genomes of various organisms, the task of identifying functional relationships between genes and molecular mechanisms of reaction of the organisms to the impact of external and internal factors becomes the most crucial issue. The analysis of transcriptome data (RNASeq, microarray expression analysis, real-time PCR, etc.) often serves as the main experimental method to establish the effect of different perturbations on gene expression. Among the most commonly used databases containing information about the level of gene expression in different tissues of human and model animals we can mention Allen Brain Atlas [1], GENSAT (Gene Expression Nervous System Atlas) [2], BGEM (Brain Gene Expression Map) [3] and GEO (Gene Expression Omnibus) [4]. Particular attention should be given to the Allen Brain Atlas (ABA) database, which provides detailed data on gene expression in various tissues of the human and mouse brain, distributed over voxels or extended regions.

Predictions of the effects of various factors on gene expression are usually performed by means of mathematical models. There are well-developed approaches to modelling the functioning of molecular genetic systems and gene networks, for example, kinetic modelling [5-7]. These models require information about the interaction parameters and reaction rates, the number of which is growing rapidly with an increase in gene network size. It should be noted that the quantitative information about the reaction rates is absent for most genes and for their products involved in the reactions. One of the most interesting areas of molecular-genetic interactions is genetic regulation. In connection with this, a large number of works presented in the literature are devoted to the study of the quantitative characteristics of genetic regulation using expression data [5,8-16]

In order to evaluate the kinetic parameters of genetic regulation, the main source of data are time-series measurements of gene expression, including RNA and protein concentrations [10-13]. However, obtaining the data on the dynamics of expression, which can be used to develop mathematical models to describe the functioning of various molecular-genetic systems is often complicated due to the methodological difficulties and high costs [12]. For this reason, when creating the mathematical models, researchers are usually faced with the inverse problem when the number of samples or dimensions in the data is smaller than the number of parameters (so-called ill-posed inverse problem). Another problem should be also highlighted that occurs when one tries to create a mathematical model that describes the actual biological processes, distributed among different tissues and organs of a body. This is a classical problem of heterogeneity of the modelled objects. In particular, the spatial heterogeneity of the gene expression in the brain is observed in the analysis of expression data from the database Allen Brain Atlas.

An alternative to using the kinetic data would be to use information about the steady-state expression levels obtained under different conditions or in different tissues [14-16]. Such type of data is more widely available in databases compared to the kinetic data and allows researchers to compute the results in terms of tissue- or condition-specific effects.

Approaches that use these data to reconstruct the network of genetic regulation and to define its parameters include approaches that use Bayesian networks [17-20] and ones based on mutual information [21,22]. Those are well-designed techniques that can identify regulatory interactions from expression data. Although these methods make it possible to generate semi-quantitative predictions, e.g. direction and relative magnitude of regulatory influences, it is difficult to obtain quantitative predictions based on models reconstructed using these methods.

Another example would be the use of machine learning techniques, including regression models, neural networks, support vector machines and others. Models built using these techniques allow quantitative predictions of the expression levels of the specific genes considering the expression levels of genes interacting with these specific genes.

However, the problem of dimensionality or completeness of the data remains in this case as well. Therefore, a number of approaches are being used to reduce the dimensionality of the model that reconstructs molecular genetic interactions from expression data. One of the approaches is to limit the number of interactions for a given gene [5,8]. Another approach to the reduction of the number of genes being analysed is to combine genes into groups based on their expression profiles in order to examine the so-called co-regulated gene modules [14,23,24]. Yet another approach is to consider only known transcription factors as independent variables in the regression equation [8,14,24].

The aim of this work was to construct a statistical model describing the potential knockout effect of target genes on the expression of genes involved in the regulation of apoptosis in various areas of the human brain. Evaluation of tissue-specific effects of a particular gene knockout on apoptosis, or programmed cell death, is a very challenging and very important question in modern pharmacology, medical systems biology and biomedicine.

It is known that apoptosis plays an important role in various biological processes including functioning of the immune system, normal cell turnover, embryonic development and others [25]. Neuronal apoptosis in the embryonic brain is significant for normal development and functioning of nervous system. Disruptions in apoptosis during embryonic development may lead to brain neuroanatomic abnormalities [26]. Moreover, dysregulation of apoptotic pathways can lead to development of pathological conditions in brain such as cancers, ischemic and autoimmune abnormalities, and neurodegenerative disorders, including Alzheimer's and Parkinson's diseases [25,27,28]. Better understanding of the molecular-genetic mechanisms involved in apoptosis regulation in the brain can help in the identification of new potential therapeutic targets for drugs against such serious diseases as cancers, Alzheimer's and Parkinson's diseases.

Using STRING [29] and ANDSystem [30] databases we reconstructed target-centric gene networks associated with genes involved in the «apoptotic process» Gene Ontology (GO) [31,32] category. Gene Ontology Annotations [31] is a constantly updated database that contains a structured, precisely defined, controlled vocabulary for describing the roles of genes and gene products in biological processes. Database of Gene Ontology Annotations is one of the most comprehensive sources of information on the relationship of genes / proteins with different biological processes, for example, about 2 thousands of genes / proteins are involved in the GO category «apoptotic process» and 213 children GO categories, associated with apoptosis. For comparison, the KEGG database includes information about 85 genes / proteins that are involved in apoptosis. Of these, 56 genes (66%) are on the list of genes associated with apoptosis according to Gene Ontology Annotations.

STRING is one of the most widely used resources in the reconstruction of molecular genetic networks. STRING database describes different types of relationships between genes, including protein-protein interactions, associations, etc., however, the genetic regulation is not explicitly presented. Therefore, in addition to the reconstruction of gene regulatory networks, we used a system developed earlier called ANDSystem, containing information about interactions between genes, proteins, metabolites, biological processes and diseases that is automatically extracted from scientific publications. Interactions between molecular genetic entities in ANDSystem are classified into more than 20 different types of interactions, which includes both positive and negative regulation of the gene expression by products of other genes, as well as regulation of unknown sign. Using information about the direct and indirect relationships between genes in the reconstructed gene network has allowed us to significantly reduce the dimensionality of the model, providing an opportunity to train the model on the available experimental data. Our study examined 12 major anatomical structures of the brain, as well as 1,900 genes related to apoptosis (objective genes) according to the Gene Ontology Annotations [31] database, and more than 7,500 target genes, for which we found a link to one or more of objective genes and analysed their corresponding knockout effect in our model. It turned out that among the examined target genes some showed a pronounced structure-specific effect of their knockout on the expression of objective genes in distinct anatomical structures of the brain according to created models. The analysed target genes included the genes directly involved in the «apoptotic process» Gene Ontology biological process. But a significant number of target genes were also presented by new potential pharmacological targets that are not included in the list of the apoptosis genes.

Gene Ontology enrichment analysis was conducted on a set of target genes with the highest values of calculated knockout effects on the apoptosis network and additionally showed the importance of several Gene Ontology categories, such as the regulation of metabolic processes, cell proliferation and cell death, immune processes, response to various stimuli and stress.

Genes, expression downregulation of which has a predicted effect on apoptosis, can be considered as potential pharmacological targets. In this regard, it was important to analyse the distribution of known drug targets among genes with the highest predicted knockout effect values on the apoptosis network in different regions of the brain corresponding to distinct anatomical structures. The analysis showed that among the potential target genes that have the strongest effect on apoptosis, there are a number of known pharmacological targets. In particular, the percentage of known targets was increasing with the calculated knockout effect value. Interestingly, among the known pharmacological targets the genes were identified that were known targets for drugs against cancer and immune system diseases.

Comparison of the predictions of target genes knockout effect on apoptosis with the experimental data on siRNA-inhibition [33,34] showed good agreement between the theoretical and experimental data. Genes with experimentally confirmed connection between their expression suppression and caspase 3 activation, as well as genes that significantly induced cell death upon their downregulation in T98G glioblastoma-derived cell line, had significantly higher knockout effect calculated using our models compared to average effect among analysed target genes.

Thus, the developed method can be used in evaluating the effect of inhibition of specific genes on the function of the specific biological processes in different tissues and structures of the brain. The obtained results of target genes knockout effect considering their spatial distribution among brain structures on the programmed cell death, can serve as the basis for the search for pharmacological targets with enhanced effects in the specifically selected brain areas.

## Methods

For creating statistical models, we used expression data available from the Allen Brain Atlas. Those data were used as "initial" experimental values. The data contained whole-genome normalized microarray expression data for different anatomical areas of adult human brain. Allen Brain Atlas contained human brain expression data for six different donors with data on various brain areas for different donors. All donors were with no known neuropsychiatric or neuropathological history, no history of long-lasting hypoxic conditions, and no infectious diseases found by a serology screen. Detailed data acquisition and normalization procedures are described in [35]. Brief analysis showed that whole brain average gene expression values were highly correlated between donors (Pearson correlation coefficient >0.95), therefore it was decided to use data for H0351.2002 donor, which contained information on the largest number of different areas of the brain (893).

Allen Brain Atlas data included repeated measurements for a number of genes and brain regions, and for each region of the brain its spatial coordinates on the MRI image were included. Those coordinates were further used when presenting images of spatial distribution of calculated values in the brain. For each brain area the Allen Brain Atlas also provided corresponding anatomical brain structure. This information was used to predict the effect of a knockout of target genes on the expression of objective genes in given brain structures. During the preliminary processing the repeated measurements were averaged and the resulting normalized data for expression levels of 18,242 genes in 893 spatial areas of the brain were obtained.

When constructing the models we used the genes involved in «apoptotic process» GO category and all its children GO categories as genes of interest (further, objective genes), i.e. we modelled effect of gene knockouts on expression of those genes only. All the genes interacting with the objective genes were analysed as potential knockout targets (further, target genes). To build gene networks that describe direct and indirect interactions between target and objective human genes we used STRING [29] and ANDSystem [30]. STRING is a well-known database of interactions between genes/proteins accumulating a large number of information sources, including factual and supervised databases. In the STRING database each link between genes is described with a combined weight (combined score), which is a parameter that determines the statistical significance of relationships. In this paper, the threshold for the combined weight was chosen as 0.9, corresponding to the highest degree of reliability (highest confidence, as described in STRING database). ANDSystem was previously developed for the automatic extraction of knowledge about molecular-genetic interactions between proteins, genes, metabolites, biological processes and diseases from the texts of scientific publications and their presentation in the form of associative semantic networks. All interactions in ANDSystem are divided into more than 20 categories. Unlike STRING database the ANDSystem explicitly describes interactions related to gene regulation, including positive and negative regulation of gene expression. In this regard, ANDSystem was used to reconstruct gene regulatory networks which included only interactions corresponding to gene expression regulation.

Gene Ontology biological processes enrichment analysis was performed using the BiNGO plugin of Cytoscape system [36,37]. Benjamini & Hochberg False Discovery Rate (FDR) correction was used. In addition to standard enrichment analysis we also performed an analysis of functional connectivity of target genes considered in the enrichment analysis. We assume that the connectivity of genes involved in a common biological process exceed the connectivity between randomly selected genes. Earlier, we used the approach in the analysis of molecular association of pathogenetic contributors to pre-eclampsia [38], as well as in the analysis of molecular genetic mechanisms of dystropy [39]. It was shown that functionally connected genes have more connections between each other than randomly selected genes. Therefore, evaluation of the functional connectivity between genes that determine overrepresented GO processes can be used to detect GO processes most closely associated with the analysed set of genes.

To assess the functional connectivity of a set of target genes involved in a given overrepresented GO category we compared the gene network with these genes as vertices with networks built on random sets of genes. For each overrepresented GO category we automatically created 1,000 gene networks with random sets of genes using ANDSystem. Random sets of genes were chosen to be of the same length and having the same node degree distribution as the set of analysed target genes from the GO category thus ensuring the adequacy of further comparison. For each analysed target gene we formed the restricted list consisting of genes with the same vertex degree as for target gene. To avoid a connectivity bias related to the possible presence of target vertices with high degrees in contrast with random vertices we restricted the list of genes from which we randomly take a vertex. Then, we randomly took a vertex from this list to reconstruct a semi-random network. After the reconstruction of the networks the distribution of the number of links was built for the semi-random networks. As the characteristic of functional connectivity between genes in the gene network constructed by genes directly involved in the GO category, we considered the frequency of observing the semi-random networks with equal or greater number of connections. This approach assumes that functionally related genes have a greater number of interactions with each other than genes from a random set. Calculated in this way the measure of functional connectivity was used to rearrange the significantly overrepresented GO biological processes.

Visualization of the spatial distributions of the obtained values for each spatial area was performed using the nilearn [40] library of the sci-kit learn [41] Python package. The spatial coordinates of brain areas were taken from Allen Brain Atlas database. Maximum intensity projection (MIP) [42] method was utilized in order to present three-dimensional distributions on paper. Briefly, this method presents as a 2D image a projection of maximum intensities of the original 3D distribution along the direction orthogonal to the image plane.

When comparing top-100 lists of genes for different brain structures we performed hierarchical clustering and dendrogram visualization using linkage and dendrogram functions of "hierarchy" module of sci-kit learn [41] Python package. Pairwise distances between analysed lists were calculated as a number of mismatches between them (size of union minus size of intersection).

Information about the genes that are known pharmacological targets was extracted from DrugBank database [43]. This information was used to search for known pharmacological targets among potential target genes predicted by the analysis of constructed models. Information about drugs and related diseases was taken from Kyoto Encyclopedia of Genes and Genomes (KEGG) [44,45].

## Results and discussion

### Development of target-centric statistical models describing the effect of the inhibition of the target gene on the network of objective genes

Creating a statistical model of the effect of inhibiting the target gene on expression of genes from the objective gene network encompasses two main steps. In the first step, the target-centric gene network is reconstructed, describing the interaction of the target gene with the objective genes that are its immediate neighbours, and also all direct connections of such objective genes. Let *T *denote the target gene, $N_{j}^{T}$ - the objective genes that are neighbours of a target gene (*j=*1*..m_T_*, where *m_T _*is the number of objective genes linked to *T*), and all the neighbours of objective genes $N_{j}^{T}$ let in turn be $N_{i}^{N_{j}^{T}}$. It should be noted that *T *itself is included as one of $N_{i}^{N_{j}^{T}}$.

**Figure 1:**
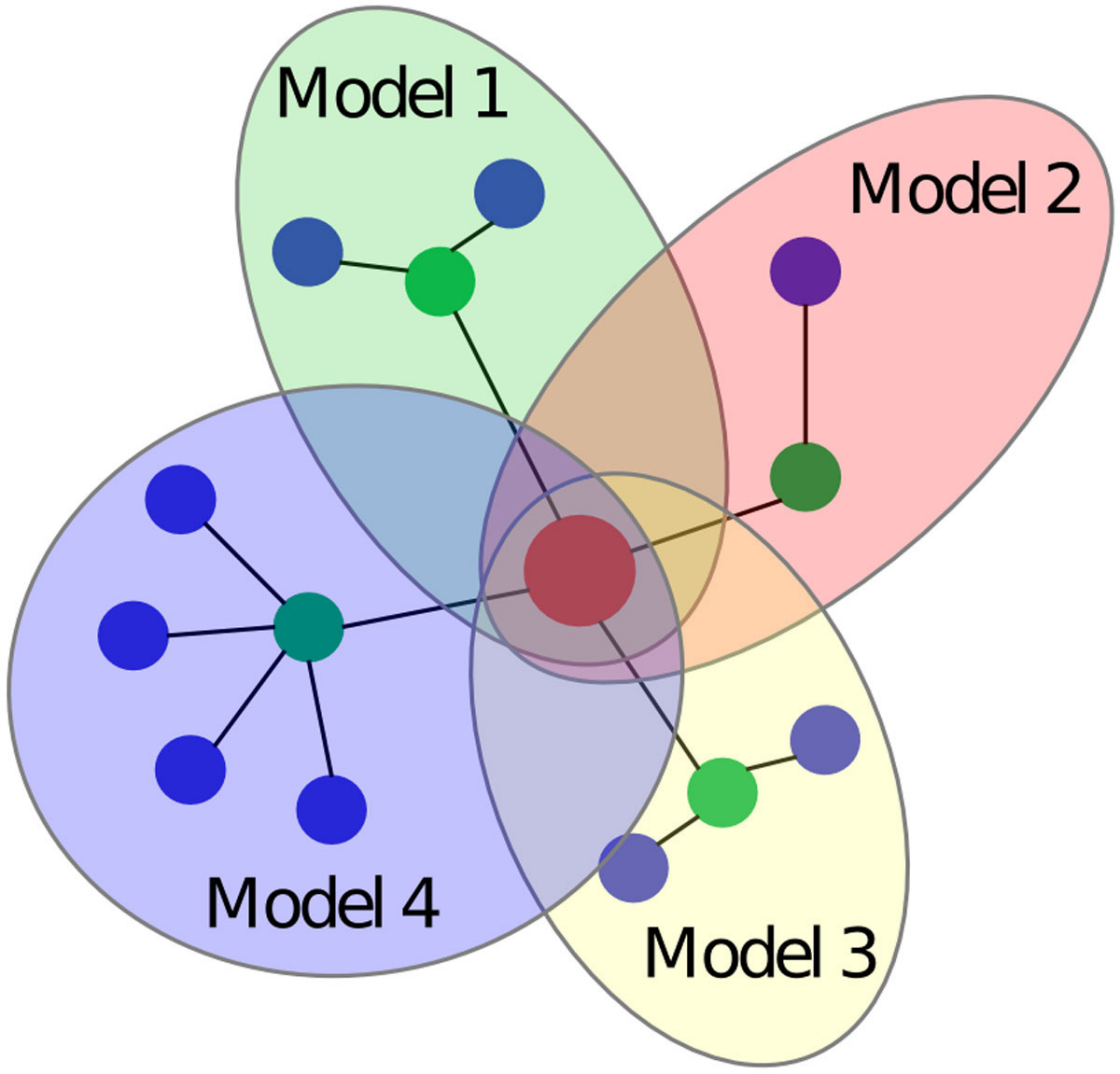
**Schematic representation of the target-centric gene network**. The target gene (T) is shown with a red circle, green circles show the objective genes $N_{j}^{T}$, which are the direct neighbours of the target gene, blue circles denote the neighbours of target genes $N_{i}^{N_{j}^{T}}$. Edges denote interactions between genes. Coverage of regression models is shown with coloured ellipses.

A schematic representation of target-centric model is presented on Figure 1. For example in case when considering *Nod1 *as a target gene the corresponding target-centric network contained 219 nodes. The *Nod1 *gene codes a cytosolic protein that recognizes bacterial molecules and stimulates an immune reaction and is known to participate in caspase-9 and NF-κB activation [46,47]. For this gene *m_T _*= 6, i.e. there were 6 neighbours from "apoptotic process" GO category ($N_{j}^{T}$), which were CASP1, CASP8, CASP9, CARD6, CCK and RIPK2. Number of neighbours ($N_{i}^{N_{j}^{T}}$) for each of them was 27, 56, 25, 3, 141 and 38, respectively. These neighbours included the target, *Nod1*. Due to some amount of intersection (i.e. some $N_{j}^{T}$ had similar neighbours or were neighbours of each other) the total number of unique nodes in the analysed target-centric network in this case was 219, which is less than the sum of numbers of neighbours, 290.

In the second step for each objective gene $N_{j}^{T}$ a regression model is built where the expression level of the objective gene $N_{j}^{T}$ serves as dependent variable, and the independent variables are the expression levels of its immediate neighbours $N_{i}^{N_{j}^{T}}$. To establish the connection between the levels of expression of the objective gene and the target gene we selected multiple linear regression analysis, as the initial stage of work had the task of implementing and evaluating the simplest models. The regression equation can be written as follows:

(1) $$E_{p}\left(N_{j}^{T}\right)=\underset{i}{\sum }k_{j}^{i}E_{p}\left(N_{i}^{N_{j}^{T}}\right)+k_{j}^{0},$$

where $E_{p}\left(N_{j}^{T}\right)$ is the expression level of objective gene $N_{j}^{T}$ in brain area *p*, summation is over neighbours of the objective gene $N_{j}^{T}$, $E_{p}\left(N_{i}^{N_{j}^{T}}\right)$ is the expression level of $N_{i}^{N_{j}^{T}}$ neighbour of the objective gene $N_{j}^{T}$ in brain area *p*, and $k_{j}^{i}$, $k_{j}^{0}$ are the regression coefficients to be determined from expression data for different brain areas *p*.

Thus, for each target gene *T *we built a series of regression models, one for each objective gene $N_{j}^{T}$ interacting with *T*. Each of those regression models described how the expression level of $N_{j}^{T}$ depends on the expression levels of $N_{i}^{N_{j}^{T}}$ genes (genes that directly interact with $N_{j}^{T}$, including original target *T*). In this case the dependency was described using linear model with corresponding coefficients $k_{j}^{i}$, $k_{j}^{0}$. For example, a target-centric model for previously mentioned *Nod1 *gene included six regression models corresponding to six objective genes that are neighbours of *Nod1 *gene in the gene network.

As a training sample to determine the regression parameters we used data on spatial distribution of gene expression levels in different parts of the human brain, presented in the Allen Brain Atlas database. Prediction of changes in expression levels of each of the *m_T _*objective genes after a knockout of the target gene was carried out using regression models corresponding to the objective genes. In such models, the target gene is one of the independent variables. To simulate a knockout, the expression level of the target gene was set to zero and expression values of objective genes were recalculated using models from Eq. 1. In case of simulation of the effect of activating a target gene, its expression level values may be increased to the desired value.

The effect of the resulting target gene knockout $R_{p}^{T}$ for a given spatial area *p *is calculated as the sum of moduli of relative changes in expression levels of the objective genes for this target. The change is calculated between the initial values of expression and the values calculated for the knockout of the target gene, as follows:

(2) $$R_{p}^{T}=\overset{m_{T}}{\underset{1}{\sum }}\left|\frac{E_{p}^{0}\left(N_{j}^{T}\right)-E_{p}\left(N_{j}^{T}\right)}{E_{p}\left(N_{j}^{T}\right)}\right|,$$

where $E_{p}\left(N_{j}^{T}\right)$ is the initial expression value of $N_{j}^{T}$ in brain area *p *and $E_{p}^{0}\left(N_{j}^{T}\right)$ is the corresponding expression level predicted for the knockout of target gene *T *and the summation is over objective genes interacting with *T*.

Since the aim of this paper was to analyse changes in the expression level of objective genes after the knockout of the target gene regardless of the direction of this change, the modulus is used in the calculation of $R_{p}^{T}$. To take into account the direction of changes in the expression levels a different index should be used. To assess the effect of a target gene knockout on the expression of objective genes in a certain predetermined brain structure *s*, we calculated the change in levels of expression of the objective genes $R_{s}^{T}$, by averaging $R_{p}^{T}$ for all spatial points of the brain structure *s*.

The specificity index of the knockout effect to the brain structure s was calculated as a fold change:

(3) $$FC_{s}^{T}=\frac{R_{s}^{T}}{\frac{1}{NS-1}\sum_{d\neq s}R_{d}^{T}},$$

where *NS *is the total number of structures in the brain, and the denominator is a sum over all the brain structures but *s*. This indicator reflects the ratio of changes in the expression levels of all objective genes in the structure *s *to the changes averaged over the remaining brain structures.

$FC_{s}^{T}$ value clearly reflects the structure specificity of the knockout effect of the target gene on the expression of objective genes, but it has a drawback. $FC_{s}^{T}$ may have an extremely high value for a particular structure *s*, i.e. corresponding effect demonstrates high spatial specificity, when simultaneously the underlying effect can be insignificant (low value of the $R_{s}^{T}$ index).

To solve this problem, we introduced yet another indicator of rank specificity (RankSpec), which is calculated as the average ranks of a given gene in the lists of target genes, sorted by values of $FC_{s}^{T}$ and $R_{s}^{T}$ parameters. The same approach of average rank can be seen, for example, in [48] and it is used to rank objects based on several criteria simultaneously. For convenience, the range of RankSpec values was set from 0 to 1 by normalizing $R_{s}^{T}$ and $FC_{s}^{T}$ ranks to their corresponding maximum values.

In addition, to estimate the average effect of a knockout on the whole brain without dividing it into different anatomical structures we introduced $R_{all}^{T}$ index, which was calculated as the average value of $R_{p}^{T}$ among all 893 spatial areas of the brain.

To estimate the effect of the knockouts specifically on apoptosis, we only considered genes involved in «apoptotic process» GO category as objective genes.

### Analysis of the structure-specific knockout effect of target genes on the expression of objective genes involved in a GO category "apoptotic process"

List of genes involved in the «apoptotic process» GO category included more than 1,900 human genes. Using the STRING database we built more than 6,500 target-centric networks in which the target gene contained at least one neighbour from the family of the apoptotic genes. Using the ANDSystem database more than 4,000 target-centric networks were built which included only genetic regulation relationships as links.

**Figure 2:**
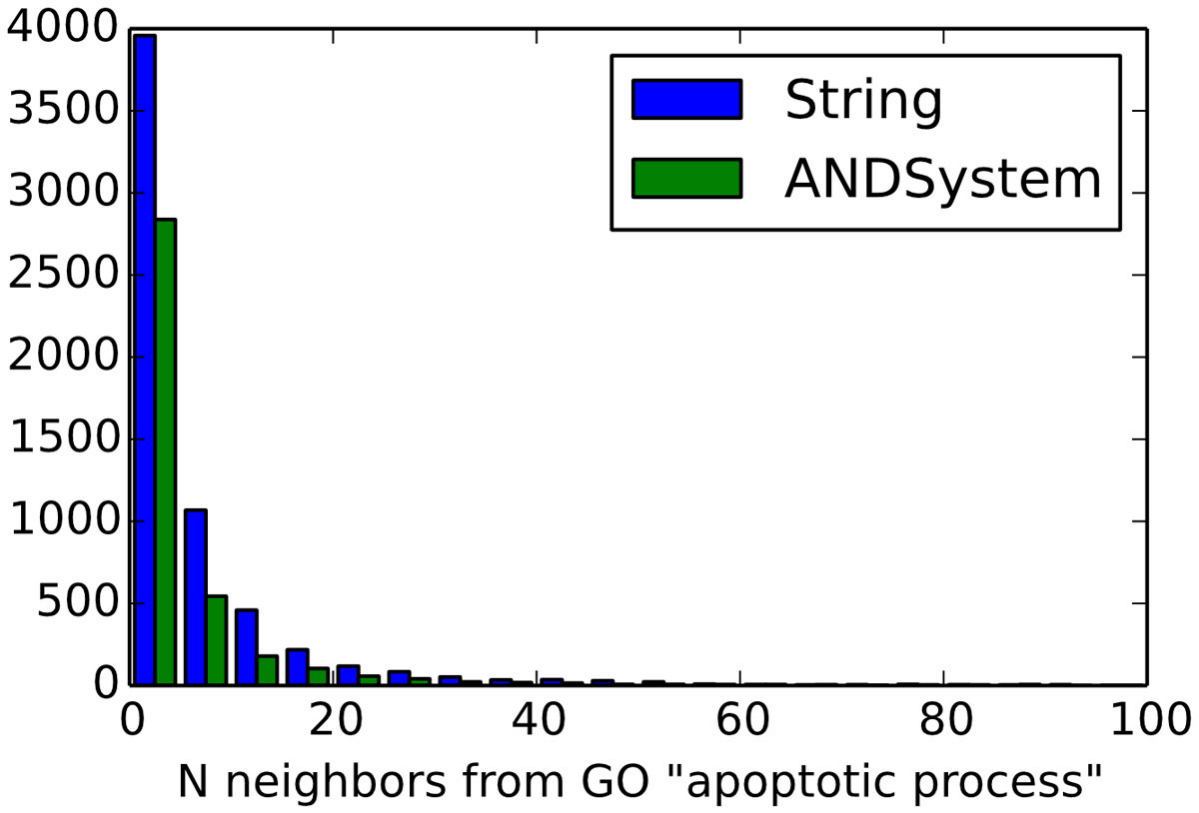
**Distribution of the number of connections of target genes with objective genes**. Diagram presents the distribution for objective-centric networks reconstructed using STRING (shown in blue) and ANDSystem (shown in green) databases.

Distribution of the number of connections of target genes with objective genes is presented in Figure 2. As expected for the node degree distributions of biological networks [49], it turned out to be the power-law distribution. As can be seen, the maximum number of target-centric networks contained only one objective gene linked to the target gene. In the set of networks built using STRING database more than 140 networks contained more than 100 objective genes linked to a target gene, whereas for ANDSystem there were only 15 such networks.

For each target-centric network a set of objective-centric models was constructed using multiple linear regression analysis to predict the effect of a target gene knockout on the expression of the objective genes and, as a result, on the regulation of apoptosis. Training of each model was performed on expression data for 893 spatial areas of the brain. To investigate the spatial distribution of the knockout effect of target genes on the expression of objective genes, we examined the main anatomical structures in a hierarchy represented in base Allen Brain Atlas. A total of 12 corresponding to upper hierarchical classification categories were selected (Table 1).

**Table 1:** Knockout effect values $R_{s}^{T}$ of 8 top target genes on expression of objective genes involved in "apoptotic process" GO category.

Brain structures	UBC *	RAC1 *	PTPN11	CDC42 *	FYN *	LAT2	CTNNB1 *	GRB2
Whole brain**	105.0 **/ 0.95**	48.4 **/ 0.44**	31.3 **/ 0.28**	29.3 **/ 0.27**	27.5 **/ 0.25**	26.5 **/ 0.24**	26.3 **/ 0.24**	24.3 **/ 0.22**
Cerebellum	110.2 **/ 1.00**	50.8 **/ 0.46**	30.2 **/ 0.27**	32.6 **/ 0.30**	30.0 **/ 0.27**	26.2 **/ 0.24**	29.9 **/ 0.27**	26.5 **/ 0.24**
Pons	104.5 **/ 0.95**	49.3 **/ 0.45**	31.3 **/ 0.28**	32.6 **/ 0.30**	27.0 **/ 0.25**	28.7 **/ 0.26**	25.0 **/ 0.23**	22.9 **/ 0.21**
Thalamus	102.5 **/ 0.93**	49.8 **/ 0.45**	31.8 **/ 0.29**	32.5 **/ 0.29**	28.0 **/ 0.25**	27.4 **/ 0.25**	26.3 **/ 0.24**	23.2 **/ 0.21**
Hypothalamus	103.1 **/ 0.94**	48.9 **/ 0.44**	30.8 **/ 0.28**	31.6 **/ 0.29**	27.2 **/ 0.25**	27.5 **/ 0.25**	28.1 **/ 0.25**	23.5 **/ 0.21**
Basal ganglia	104.2 **/ 0.95**	48.5 **/ 0.44**	33.1 **/ 0.30**	28.7 **/ 0.26**	26.6 **/ 0.24**	28.3 **/ 0.26**	26.9 **/ 0.24**	24.0 **/ 0.22**
Amygdala	103.8 **/ 0.94**	47.2 **/ 0.43**	31.9 **/ 0.29**	29.1 **/ 0.26**	28.0 **/ 0.25**	27.1 **/ 0.25**	27.6 **/ 0.25**	24.1 **/ 0.22**
Hippocampus	103.8 **/ 0.94**	51.2 **/ 0.46**	31.8 **/ 0.29**	31.2 **/ 0.28**	29.3 **/ 0.27**	26.4 **/ 0.24**	28.3 **/ 0.26**	25.6 **/ 0.23**
Cingulate gyrus	105.7 **/ 0.96**	46.9 **/ 0.43**	30.8 **/ 0.28**	27.2 **/ 0.25**	27.1 **/ 0.25**	25.1 **/ 0.23**	24.7 **/ 0.22**	23.9 **/ 0.22**
Temporal lobe	105.5 **/ 0.96**	46.7 **/ 0.42**	30.9 **/ 0.28**	26.6 **/ 0.24**	26.9 **/ 0.24**	25.2 **/ 0.23**	24.8 **/ 0.23**	24.6 **/ 0.22**
Parietal lobe	105.2 **/ 0.95**	47.0 **/ 0.43**	31.0 **/ 0.28**	26.7 **/ 0.24**	26.6 **/ 0.24**	25.2 **/ 0.23**	24.7 **/ 0.22**	24.3 **/ 0.22**
Frontal lobe	106.0 **/ 0.96**	47.3 **/ 0.43**	30.9 **/ 0.28**	27.0 **/ 0.25**	26.5 **/ 0.24**	25.5 **/ 0.23**	24.2 **/ 0.22**	24.4 **/ 0.22**
Occipital lobe	105.7 **/ 0.96**	46.9 **/ 0.43**	31.6 **/ 0.29**	26.2 **/ 0.24**	26.6 **/ 0.24**	25.2 **/ 0.23**	24.7 **/ 0.22**	24.6 **/ 0.22**

Each cell contains original $R_{s}^{T}$ value and $R_{s}^{T}$ divided by maximum of $R_{s}^{T}$ along all structures all genes, separated by a slash.

* target genes involved in "apoptotic process" GO category

** $R_{all}^{T}$

The accuracy of each objective-centric regression model was estimated using cross-validation. Gene expression data included in the model was randomly divided into two subsets, one of which containing 90% of the original spatial areas was used to train the model, while the other (a test set containing the remaining 10% of the points), was used to evaluate accuracy of the model. The procedure was repeated 1,000 times, after which the average value of the root mean square of difference between the predicted and experimental values of gene expression of the objective gene for the spatial points of test sample, divided by experimental values, was calculated and used as a relative error value for the model.

The average error of the models was 9% and 11% for STRING and ANDSystem networks, respectively. Distribution of error was asymmetric with elongated tail on the right side. The nature of error distributions did not significantly differ between STRING and ANDSystem models. Higher error rates could be explained by the possible non-linear nature of expression regulation which could be caused by the effects of epigenetic control. Another possible reason for the errors is the incompleteness of relationships considered in the gene network.

To ensure the reliability of the results, models with error rate of more than 25% were excluded from further analysis. After exclusion, 6,133 and 3,880 target-centric knockout models remained for STRING and ANDSystem networks, respectively. These models were used to assess the knockout effect of target genes on the expression of objective genes. Due to the mismatch between gene networks, the total of 7,540 different target genes was considered.

For example, Table 1 shows the values of structure-specific knockout effect values $R_{s}^{T}$ of the top 10 target genes for each of the 12 anatomical structures. These genes had the strongest knockout effect on the objective genes $R_{all}^{T}$, i.e. averaged over all 893 spatial areas of the brain. The tabulated knockout effect values were calculated using STRING models. Interestingly all of these genes do not show a pronounced specificity to any anatomical structure. Average effects of these genes on different structures were approximately equal, and relative spread of $R_{s}^{T}$values did not exceed 25%.

Target genes with the highest values of structure-specificity of the knockout effect (RankSpec) are shown in Table 2. From the table it can be seen that the gene *NR3C1 *has a maximum specificity to the cerebellum, i.e. the suppression of its expression leads to the effect on the expression of objective genes of "apoptotic process" GO category which is highly specific to this structure. *NR3C1 *gene encodes the glucocorticoid receptor, which is a transcription regulator of other genes, including those involved in the regulation of cell death and apoptosis [50,51]. It is known that glucocorticoid receptors play a key role in the hypothalamic-pituitary-adrenal (HPA) axis, which is important in the stress response [52]. It has been shown that patients who survived early psychosocial stress, as well as a wide range of mental disorders, show change in the patterns of *NR3C1 *methylation and dysregulation of HPA axis. Furthermore, in Kitraki et al., 1999 [36] it has been shown that chronic stress causes a down-regulation of glucocorticoid receptor mRNA in cerebellum and hippocampus in rats.

**Table 2:** Target genes with highest RankSpec values for different anatomical structures of the brain.

Structure	Rank	1	2	3	4	5	6	7	8	9	10
cerebellum		NR3C1*	PAN2	CYTSA	DPYSL2	GABRB3*	GABRG2	NPM1*	ABL2*	ACAT2	PITPNA
pons		FOXA2	ESR1*	PPARG*	NR1D2	ESRRG	NTN1*	UNC5B*	THRA*	NR4A2*	FOXO1*
thalamus		DPYSL2	FOXA2	FOXO1*	PITPNA	CRMP1	PTGDS	RORA	CDK5*	DPYSL4	MCAT
hypothalamus		FOXA2	FOXO1*	NFATC1	POMC	NR2F2	NKX2-2	TBXA2R	TACR2	FZD10	NTS
basal ganglia		FOXO1*	FOXA2	TPM1	SYN1	GAD2	MCM7	LRP6*	ACTN2*	MCM3	SST*
amygdala		MYB	NPY1R	NR2F2	NPY	PNOC	PDYN	ZNF32	ATF1	RGS7	FOXO1*
hippocampus		DPYSL2	CRMP1	PRKCA*	CDK5*	PA2G4*	DCC*	NOP58	DPYSL4	ERBB2	WNT3A*
cingulate gyrus		SQSTM1*	AES*	NEDD4L	PITPNA	ADCY2	SHC2	BTRC	NTRK2*	ZFPM1	MEF2C*
temporal lobe		AES*	ADCY2	SQSTM1*	NEDD4L	KIDINS220	STAT6	SHC2	NTRK2*	MEF2C*	CCK*
parietal lobe		AES*	STAT6	SQSTM1*	ADCY2	KIDINS220	NEDD4L	MEF2C*	RPF1	ZFPM1	CCK*
frontal lobe		SQSTM1*	AES*	NEDD4L	ADCY2	SHC2	BTRC	STAT6	MEF2C*	TRAF3*	ECSIT
occipital lobe		AES*	ERBB4*	UBE2D2	MEF2C*	ADRBK1	RPF1	DNM1	PTTG1	PRKCB*	KIDINS220

* target genes involved in "apoptotic process" GO category

**Figure 3:**
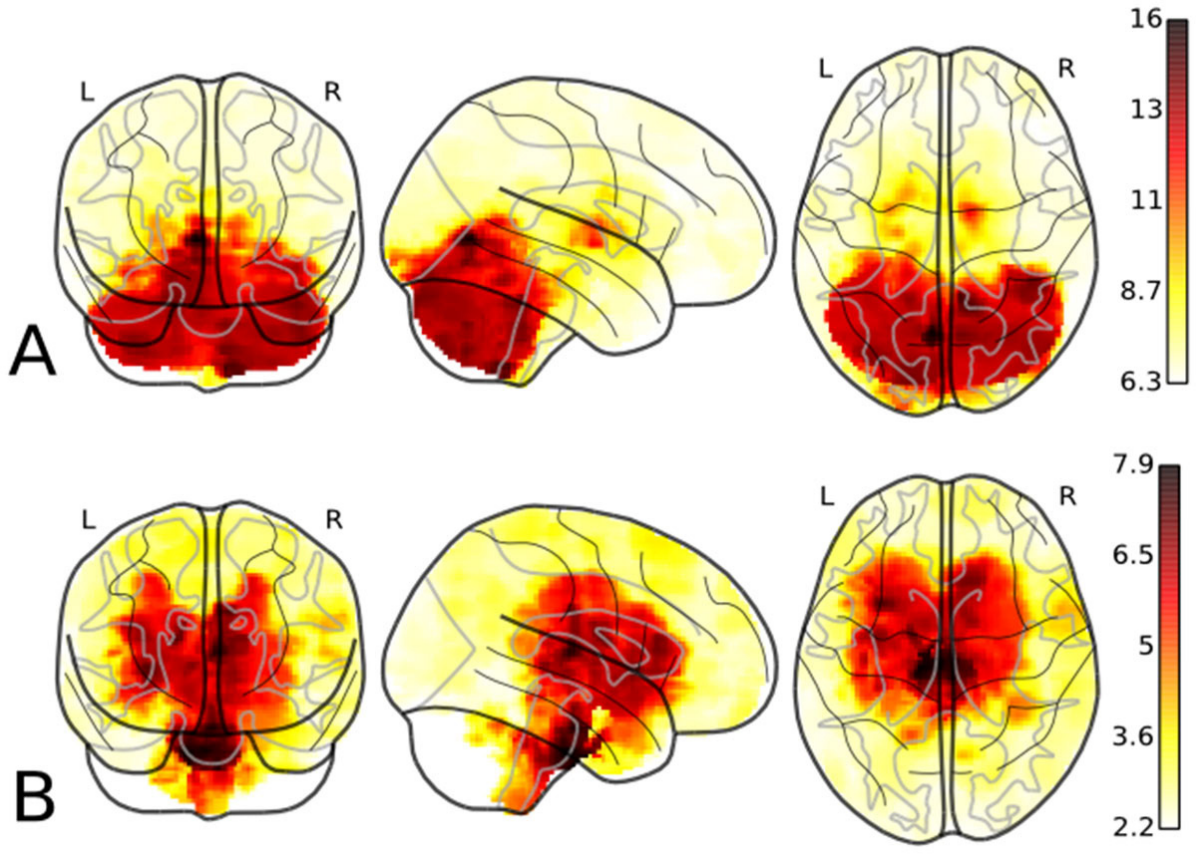
**Spatial distribution of the *NR3C1 *(A) and *Foxa2 *(B) genes knockout effects $R_{p}^{T}$**. Three orthogonal maximum intensity projections are presented. The darker regions correspond to higher $R_{p}^{T}$ values. It can be seen that the effect is concentrated mainly in the cerebellum (A) and pons (B).

Graphical representation of the distribution of the gene knockout effect intensity among different brain areas is shown in Figure 3. For example, it can be visualized for the *NR3C1 *gene (Figure 3A) that its knockout has the highest effect in the cerebellum (depicted in red) whereas it has only marginal effects in the other areas of the (depicted with a light-yellow colour). For comparison the same distribution for gene *Foxa2 *is also presented (Figure 3B). According to our results, this gene has the highest RankSpec value for pons brain structure (Table 2). The corresponding brain region is depicted in dark-red colour on the figure which corresponds to the highest values of the predicted knockout effect. At the same type, areas of cortex and cerebellum are depicted in light-yellow and white which corresponds to the lowest predicted knockout effect on the apoptosis-related genes in these areas. *Foxa2 *is a forkhead winged helix transcription factor that is involved in embryonic development and regulation of tissue-specific gene expression. It was shown that *Foxa2 *gene plays an important role in the apoptosis process [53,54] and is also a gene network regulator in several types of cancer [55,56]. Besides that, *Foxa2 *regulates differentiation of dopaminergic neurons in the brain structure adjacent to pons, the midbrain [57,58].

In order to evaluate the effect of errors in the reconstruction of gene network on the developed method, computer experiments were conducted with a random mixing of up to 10% of the connections in the gene network. It turned out that the noise had no significant effect on the $R_{s}^{T}$, the value of this effect did not exceed the noise levels in the network. The change in the index RankSpec was bigger, but the simulated noise did not change the lists of top genes shown in Table 2.

**Figure 4:**
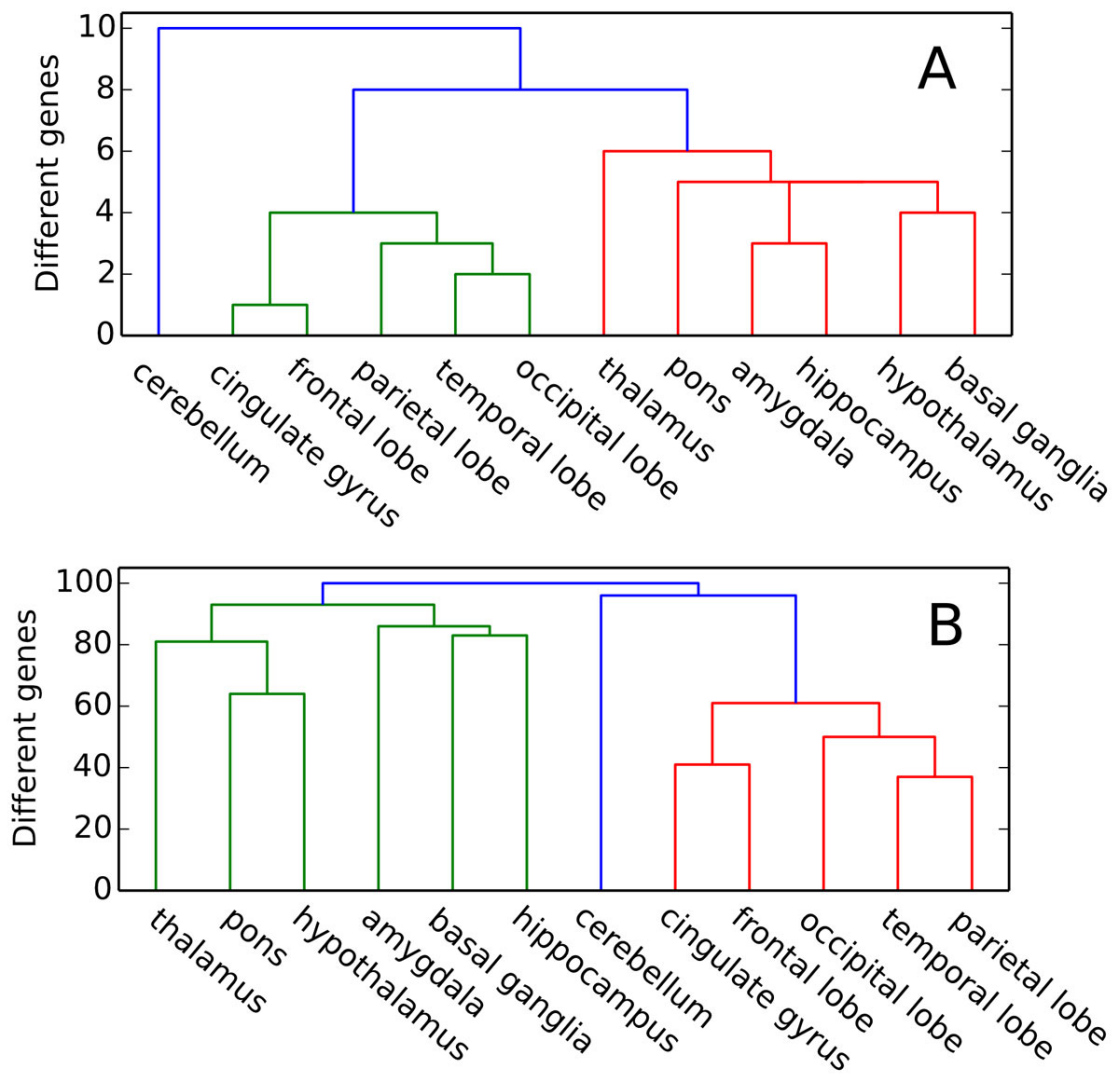
**Hierarchical clustering of brain structures based on similarity of the top 100 genes lists**. Lists of genes were ranked by knockout effect on the expression of objective genes $R_{s}^{T}$ (A) and by RankSpec values (B)

We were interested in testing the hypothesis that the gene knockout effect has similar character in anatomically close brain regions. To test this, we conducted a hierarchical clustering of brain structures by comparing the top 100 target genes in the list of target genes, ranked by knockout effect $R_{s}^{T}$ for each structure. This clustering considered two cases, the first when the ranking was conducted using $R_{s}^{T}$ effect values (Figure 4A), and the second when genes were ranked based on RankSpec effect specificity values (Figure 4B).

As noted above, the $R_{s}^{T}$ index characterizes the level of the knockout effect on a given structure s, but it does not reflect the specificity of the effect to this structure, that is, a ratio of the effect on structure of s to the effect on other structures. Whereas RankSpec index characterizes specificity. Surprisingly for us, it turned out that the hierarchical clustering trees for these two indicators have a very similar appearance. At the same time, as can be seen from Figure 4, the brain structures are divided into three groups based on hierarchical clustering: the cortex (the cluster is shown in green), the subcortical structures (shown in red), and the cerebellum. This division partly corresponds to the natural morphological division.

However, it should be noted that the direct distance values were higher in the RankSpec clustering, which suggests that the top-100 lists of genes ranked by RankSpec value contain more genes that are specific to only one of the brain structures.

### Comparison of model predictions with experimental data

For the validation of the models, we compared the predictions of the effect of suppressing the expression of target genes on the expression of objective genes from the "apoptotic process" GO category with experimental data. From the work of Ovcharenko et al. [33], we took a list of genes, siRNA-inhibition of which was found to lead to a significant activation of caspase-3.

In [33] 34 genes were found to be linked to activation of caspase-3. A total of 30 of these genes turned out to be among analysed target genes. Four remaining genes had no direct links with the genes from the "apoptotic process" GO category for either STRING or ANDSystem networks therefore they remained untested by our method.

We assumed that in the case of adequate constructed models, predicted effect on gene expression of "apoptotic process" GO category for the knockouts of the 30 experimentally discovered genes should exceed the average effect predicted for the knockouts of other target genes, for which the experiment showed no significant activation of caspase-3. Since no tissue-specific effect can be assigned to experiments provided in [33], we considered whole brain average value $R_{all}^{T}$ as a modelled knockout effect for comparison.

Comparison of distributions of values of the knockout effect of 30 genes from [33] and the whole set of 6,133 genes from STRING and 3,880 genes from ANDSystem using Mann-Whitney criterion showed a statistically significant (p <0.001) difference between distributions. In this analysis we considered STRING and regulatory ANDSystem network separately and the results was significant for both networks.

The average value of the predicted knockout effect $R_{all}^{T}$ for the 30 experimentally found genes was 4.5 and 4.4, while the average of all target genes was 0.93 and 0.86 for the STRING and ANDSystem, respectively.

Additionally, a similar analysis was performed on data on siRNA-inhibition in glioma cell lines. To do this a list of genes was taken from the work of [34]. The list consisted of 55 genes identified in siRNA screening, that significantly induced cell death in T98G glioma cells upon downregulation of these genes. Of these 55 genes we have considered 34 as target genes for the STRING network-based models and 17 genes for ANDSystem-based models. For the rest of the genes there were no direct links with the genes from the "apoptotic process" GO category. Comparison of distributions of predicted knockout effect values of genes from experimental work [34] and the whole set of analysed target genes showed a statistically significant difference between them (p <0.001 for the STRING network and p <0.01 for ANDSystem regulatory network, Mann-Whitney criterion was used). The mean values of the predicted effect value $R_{all}^{T}$ for the experimentally discovered genes were 3.88 and 0.98 while the average values of all target genes were 0.93 and 0.86 for the STRING and ANDSystem, respectively.

Thus, the results confirm the predictive power of our model presenting statistically higher than average values of predicted knockout effect on apoptosis for genes that have been experimentally proved to have a high impact on the system when being siRNA-inhibited. However, it should be kept in mind that the comparison was carried out with the results for siRNA-inhibition. In the future we plan to expand the validation of the method and of the produced models on the effects of various factors such as small chemical compounds, genetic variations, and others. Such studies are necessary in order to determine the applicability of the models in the design of medical products.

### GO biological processes enrichment analysis of the top target genes

For detection of biological processes that involve target genes, knockout of which leads to the most severe effect on the expression of genes from GO category "apoptotic process", the GO biological processes enrichment analysis was performed (Additional file 1). The analysis was conducted for the top 100 genes that have the strongest effect averaged over all spatial areas of the brain. Among the top ten GO categories overrepresented for genes from the String network, GO biological processes associated with the response to stimuli, immune processes, viral processes, and multi-organism processes were found. For genes from the ANDSystem regulatory network among ten most overrepresented GO biological processes categories were present related to the regulation of metabolic processes, cell proliferation and cell death, response to different stimuli and stress.

To test whether the genes involved in the identified overrepresented biological processes are functionally related to each other, for each overrepresented GO category gene networks including genes from this category as vertices were compared with networks built on random gene sets (see Materials and methods). We assumed that the more functionally connected genes have the greater number of interactions between them. The functional connectivity calculated by this method was used to re-rank significantly overrepresented GO biological processes.

After re-ranking of GO categories overrepresented for genes from the String network the list of top 10 GO categories was completely changed and included categories related to response to growth factors, signaling pathways, response to injury and other. For genes from the ANDSystem regulatory network, the list of top 10 overrepresented GO categories has not changed after re-ranking. This suggests that genes included in the top 10 overrepresented GO categories are strongly related and function in a common biological process. However, among the top 50 overrepresented GO categories, biological processes related to phosphorylation received low ranks according to the criterion of the functional connectivity. For example, the category «positive regulation of phosphorylation» ranked 11th according to Bingo and ranked 328^th ^after re-ranking by functional connectivity.

Since the initial focus of our research was on the genes from "apoptotic process" GO category, taken as objective genes, the resulting list of target genes can be enriched with genes from the same GO category. Therefore, we have conducted an additional analysis of GO biological processes enrichment for top-100 genes with the highest $R_{all}^{T}$ values, which do not belong to the "apoptotic process" GO category (Additional file 2). The results for the genes found with the STRING network showed that among the top 10 overrepresented categories some remained the same as in the results without exclusion of apoptosis-related genes. Those categories include related to response to stimuli, and to immune processes. The new categories appeared in the top 10 categories were related to regulation of cellular process. For genes found with the ANDSystem network some differences were also observed among the top 10 overrepresented categories. New categories related to positive regulation of transcription and regulation of multicellular organismal process were added, whereas cell proliferation and cell death, response to different stimuli and stress were not included in the list of top 10 categories after the exclusion of apoptosis-related genes.

It is likely that knockout of genes involved in overrepresented GO biological processes found in the analysis will also have an effect on apoptosis. Therefore, these processes can be taken into account in the evaluation of side effects that may occur after the knockout of target genes.

### Overlap between potential target genes and known pharmacological targets

We were interested in identifying potential target genes used as targets for known drugs. Our analysis showed that the DrugBank database contains information on more than 1,700 known pharmacological targets among the 7,540 analysed target genes.

**Figure 5:**
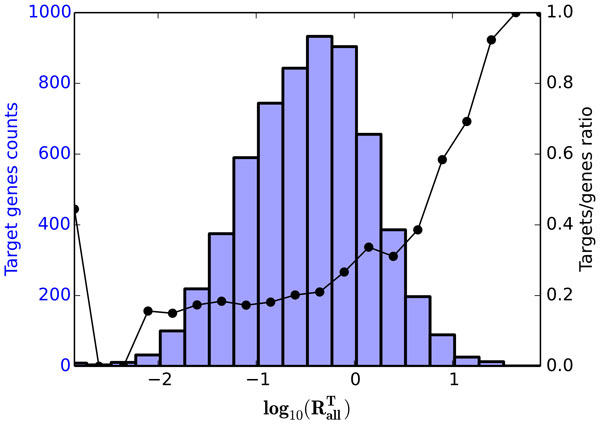
**Dependence of the ratio of the known pharmacological targets among potential target genes on the knockout effect value $R_{all}^{T}$**. Black line shows the ratio of known targets, blue histogram displays the distribution of the knockout effect values $R_{all}^{T}$. Presented are results for STRING gene network. Presented ratio approaches 1.0 at the right tail of the distribution corresponding to a small proportion of genes with the highest predicted knockout effect values.

Furthermore, it was found that the ratio of known pharmacological targets among potential target genes increases with the magnitude of the knockout effect $R_{all}^{T}$ (Figure 5). Figure 5 also shows that the highest and the lowest proportions of the known pharmacological targets fall onto the tails of the knockout effect distribution.

Unfortunately, the DrugBank database did not contain data that describes a direct relationship between pharmacological targets and diseases. However, for some of the targets the database contained data about drugs acting on these targets. To find out what diseases correspond to identified pharmacological targets, we conducted a search for the links between drugs and diseases in the KEGG database. Thus, we were able to link 204 known pharmacological targets with 112 diseases. For the rest 1,513 pharmacological targets there was no such information in KEGG.

**Figure 6:**
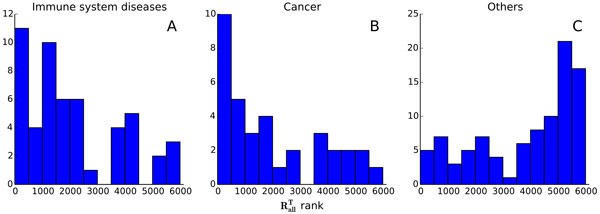
**Distribution of the knockout effects of known drug targets**. The known drug target genes are grouped by the nature of diseases the drugs are intended for: immune system diseases (A), cancer (B) and other diseases (C).

Analysis of target genes for different drugs showed that targets of drugs against different kinds of diseases have different distributions of the predicted knockout effect on apoptosis genes. All the target genes could be divided into three groups (Figure 6): targets of genes of drugs against immune system diseases (52 genes, Figure 6A), against cancer (36 genes, Figure 6B) and against other diseases (Figure 6C). The first two groups contained 43% of all known targets with identified diseases. For the first two groups, the distribution of corresponding knockout effect ranks shows that most of the target genes have higher values of effect on objective genes from the GO category "apoptotic process", whereas the same distribution for targets of drugs against other diseases (group 3, Figure 6C) shows that most of the genes have lower effects on apoptosis.

Especially important today is the study of neurodegenerative diseases. Among the potential target genes 29 turned out to be known pharmacological targets for drugs against diseases of the nervous system, including Alzheimer's disease. The targets for drugs against Alzheimer's disease included genes of nicotinic acetylcholine receptors (*CHRNB3, CHRNE, CHRNB1, CHRND*).

It is known that nicotinic acetylcholine receptors (nAChRs) play major role in the cognitive function of the brain involved in memory and sensory information processing [59-61]. Functional changes, violation of biogenesis and transport of nAChRs are important components of Alzheimer's disease [62]. nAChRs are promising targeted drugs against neurodegenerative diseases [61]. Thus, cholinergic receptor, nicotinic, beta 3 (neuronal) (*CHRNB3*) is the target of galantamine, a drug used in Alzheimer's disease [63,64]. Galantamine has a dual action on the cholinergic system: it inhibits acetylcholinesterase (AChE) and allosterically modulates the activity of nicotinic receptors (nAChRs) [64-66]. Binding of galantamine with acetylcholinesterase in the brain reduces acetylcholine (ACh) catabolism, which provides an increase in the level of acetylcholine (ACh) in the synaptic cleft. In addition to inhibition of AChE, galanthamine modulates nicotinic acetylcholine neurotransmission through allosteric potentiation of the pre- and postsynaptic nAChR [64,67,68]. Since presynaptic nAChRs may mediate the release of acetylcholine, the allosteric modulation of these receptors may increase the yield of acetylcholine and other neurotransmitters, such as glutamate and dopamine, which plays an important role in ensuring normal functioning of the brain [64,69,70].

Knockout of these genes had the greatest predicted effect on expression of gene involved in the GO category "apoptotic process", according to the RankSpec indicator, in brain regions such as the thalamus and pons for *CHRNB3 *and *CHRNE *genes, respectively. Knockout of *CHRNB1 *and *CHRND *genes had the greatest effect in the parietal lobe. It is known that nAChRs are widely represented in the thalamus, which is involved in the functioning of the limbic system which is, among other functions, responsible for normal memory functions [71,72]. In this regard, the impact of drugs, including galantamine, on nAChRs, has a positive effect on cognitive functions and provides a therapeutic effect in the treatment of Alzheimer's disease [63,64,71]. Our results are also consistent with recent studies of the role of the parietal lobe in the development of Alzheimer's disease [73,74]. At the moment, in the early stages of Alzheimer's disease structural, functional and metabolic changes are observed in the parietal lobe [73], including observed changes in the expression patterns of nAChRs [74].

## Conclusion

The study of tissue-specific gene knockout effect on various biological processes is an important task in drug development. The approach presented in this paper is based on statistical models and allows to predict the effect of changes in the expression of target genes on the expression of a list of objective genes, related to a given biological processes. With the use of this method an evaluation was conducted of the effect of gene knockout on expression of genes related to "apoptotic process" GO category in various brain structures.

The proposed method showed good agreement with the experimental data [33,34]. It was shown that the knockout of 30 genes taken from [33] and 55 genes taken from [34] had significantly stronger impact in the comparison with the rest of the target genes, which did not show a significant effect in the experiment.

Analysis of the models showed that for the 15% of target genes a pronounced structure-specific effect was observed on the expression of objective genes involved in the GO category "apoptotic process", for a variety of anatomical structures of the brain. Thus, our models may be useful for the search of target genes that provide the effect of drugs aimed at predetermined areas of the brain. Further we are planning to look for experimental confirmations of the findings of our theoretical work.

Among the 7,540 analysed target genes, 23% appeared to be known pharmacological targets. Analysis of the knockout effects of genes corresponding to these targets showed that these genes could be divided into three groups: a group of genes that are targets for drugs against cancer; a group of target genes for drugs against immune diseases and a group of target genes against other diseases. Most of the genes from the first and second groups showed strong predicted knockout effect on expression of genes from the GO "apoptotic process". In the third group, however, the largest number of genes had weakly expressed knockout effect according to our model.

Among the analysed target genes, nicotinic acetylcholine receptors (*CHRNB3, CHRNE, CHRNB1, CHRND*) were found, which are targets of drugs against Alzheimer's disease. Models for *CHRNB1 *and *CHRND *genes showed that their knockout would have the most pronounced effect on expression of apoptosis genes in the parietal lobe of the brain, and for gene *CHRNB3, CHRNE *in thalamus and pons, respectively.

Thus, the proposed approach can be used to examine the tissue-specific molecular mechanisms of drug action on known pharmacological targets, and also to identify new potential drug targets, specifically acting on predetermined areas of the brain. The developed method can also be applied to a wide range of problems associated with the study of the functioning of molecular genetic systems in different structures of the brain.

It should also be added that the method can be easily implemented and expanded to different objective gene systems to assess knockout effects. The method can also be easily modified to make use of different sources of expression data providing a wide range of further applications. Other models of machine learning can also be used in this method (neural networks, support-vector machines with different kernels, etc.) which would expand the range of modeled regulatory interactions. Further we plan to explore these options in order to advance the proposed method.

Thus, we developed a method of creating target-centric statistical models that can be potentially used for a wide range of tasks associated with the prediction of tissue-specific effects of various factors on gene expression, including the search for tissue-specific drug targets.

## Competing interests

The authors declare that they have no competing interests.

## Authors' contributions

EDP and VAI conceived the methods. EDP, OVS and VAI drafted the manuscript. EDP performed gene expression data pre-processing and generated the results on regression models of the knockout effect prediction. EST and OVS produced results on GO enrichment analysis. EST performed functional connectivity analysis. EDP and OVS performed the intersection of potential target genes and known pharmacological targets. OVS produced results on analysis of known pharmacological targets of drugs against Alzheimer's disease. VAI, EDP, INL and NAK revised the manuscript. VAI supervised the whole study. All authors have read the manuscript and approved the final version.

## Supplementary Material

Additional file 1 **GO biological processes enrichment analysis results on top 100 target genes**. The analysis was conducted for the top 100 genes that have the strongest effect averaged over all spatial areas of the brain. Click here for file

Additional file 2 **GO biological processes enrichment analysis results on top 100 target genes not included in the list of apoptotic genes**. The analysis was conducted for the top 100 genes that have the strongest effect averaged over all spatial areas of the brain, which do not belong to the "apoptotic process" GO category. Click here for file

## Declarations

Publication of this article has been funded by Russian Science Foundation grant No 14-44-00011.

This article has been published as part of *BMC Genomics *Volume 16 Supplement 13, 2015: Selected articles from the 7th International Young Scientists School "Systems Biology and Bioinformatics" (SBB'2015): Genomics. The full contents of the supplement are available online at http://www.biomedcentral.com/bmcgenomics/supplements/16/S13.
